# Level of positive mental health in the European Union: Results from the Eurobarometer 2002 survey

**DOI:** 10.1186/1745-0179-1-9

**Published:** 2005-07-21

**Authors:** Ville Lehtinen, Britta Sohlman, Viviane Kovess-Masfety

**Affiliations:** 1 National Research and Development Centre for Welfare and Health STAKES, Helsinki, Finland; 2 MGEN Public Health Research Department, René Descartes University, Paris, France

**Keywords:** Positive mental health, population survey, across-country comparisons, determinants of mental health, social support

## Abstract

**Background:**

Few epidemiological studies have focused on the occurrence of positive mental health, and those comparing several countries practically non-existent. This study presents comparative findings of positive mental health in 11 EU countries or regions, based on the Eurobarometer 2002 (autumn) survey.

**Method:**

The sample (n = 10,878) represents the general population, aged 15 or over, of 11 European countries or regions (all old EU Member States except Denmark, Greece, Ireland, Finland and Great Britain which had to be excluded because of poor response rate, less than 45%). The method of opinion survey was applied using face-to-face interviews. The Energy and Vitality Index (EVI) from the SF-36 questionnaire was use as measure of positive mental health.

**Results:**

Overall, there were between-country differences in the gender- and age-adjusted EVI mean scores. In general, poorer mental health was found in women, older age groups, those in poor economic position and those experiencing weak social support.

**Conclusion:**

Methodological biases cannot be fully excluded, and thus, one has to take the presented results with certain caution, especially when comparing the results from the different countries. On the other hand, the results on the determinants of positive mental health are in concordance with most previous studies.

## Introduction

Epidemiological population surveys, analysing determinants of positive mental health [e.g. [1-6]], are relatively uncommon, and those, comparing several countries within same survey, so far non-existing. However, giving some possibility for comparison with the present study, Veenhoven [7] has compiled a database, called World database on Happiness, that includes data on population surveys on happiness and life satisfaction from altogether 90 nations worldwide. This database includes data from 14 'old' EU Member States (all the others except UK). One problem of comparison is the correct definition of positive mental health. Happiness or life satisfaction are necessarily not the same as positive mental health, although they can be seen as essential components of the construct. More research on the epidemiology of positive mental health is evidently needed.

As a part of the standard Eurobarometer survey 58.2 a population of 16,230 people from 15 European Union Member States and 2 separate regions (East Germany and Northern Ireland) were approached by face-to-face interview between 28 October and 8 December 2002 [8]. Among other topics, the interview schedule of this specific survey included questions focusing on current symptoms of mental distress, positive mental health (experience of energy and vitality), availability of social support, and use of health services due to mental health problems.

Based on the results of this survey, the specific aims of this paper are to

- compare the level of positive mental health between 11 EU Member States or regions,

- to analyse some determinants of positive mental health in these countries.

## Method

### Sample

The twice-a year conducted standard Eurobarometer surveys cover the population of the respective nationalities of the EU Member States, aged 15 years and over, resident in each of the Member States. The basic sample design applied in all Member States is a multi-stage, random (probability) one. In each of the 17 EU countries/regions, a number of sampling points is drawn with probability proportional to population size (for a total coverage of the country) and to population density. The net sample sizes required are about 1000 per country/region, except Luxembourg (about 600) and Northern Ireland (about 300).

The response rates in the autumn 2002 survey varied from 23% to 84%. In 6 of the countries/regions (Denmark, Greece, Ireland, Northern Ireland, Finland and Great Britain) the response rate was less than 45%, and, because of that, these countries are excluded from the following analyses. The actual sample size in this study is thus 10,878.

### Measures

The measure of positive mental health in this study is the Energy and Vitality Index (EVI) of the SF-36 Health Survey instrument [9-11], as recommended by the European Commission funded project Establishment of a Set of Mental Health Indicators for European Union [12]. SF-36 measures perceived health status, and the different indexes are presented as sum scores ranging from 0 to 100. EVI includes four questions, and is supposed to act as an indicator of mental well-being [13,11]. EVI is generally considered as a feasible instrument for evaluating the positive aspect of mental health [14,15].

The psychometric properties of EVI along with the general SF-36 instrument have been estimated to be good [16,13,17]. The reliability of the SF-36 in general has been reported in several studies to be satisfactory. This includes the internal consistency (Cronbach's alpha) ranging from 0.62 to 0.96 and the test-retest coefficients ranging from 0.43 to 0.90 for a 6-month interval and from 0.60 to 0.81 for a 2 week interval. As for validity, it has been shown to correlate moderately well with other well known health measures, for example GHQ-12 [14]. The internal consistency of the EVI scale in this study was estimated separately for each country/region. The Cronbach's alpha was found to range from 0.74 to 0.87.

In measuring social support, the 3-item Oslo Social Support Scale [18] was used. The three questions cover the reported number of close friends and perceived concern and practical help from others and the sum score ranges from 3–12. Scores 3–7 are considered as poor, scores 8–10 as moderate and scores 11–12 as strong social support.

### Statistical methods

Analyses of variance, which were conducted according to the GLM procedure of the SAS 8.2 programme package, were used to compare the age- and gender adjusted means of the EVI scores. Weight adjustments were made when counting the proportions and mean scores for the whole EU. The difference between the EVI mean scores was considered as statistically significant when p < 0.01.

## Results

### Comparisons between countries

Overall, there were between-country differences in the gender- and age-adjusted EVI mean scores (ranging from 58.9 to 65.2). Four countries (Italy, Portugal, France and Sweden) presented a score that was statistically significantly (p < 0.01) lower than in at least some of the other countries. For Italy the score was lower than in all the other countries/regions; Portugal and France had a lower score than Belgium, Spain, West Germany and the Netherlands, and the score for Sweden was lower than that for Belgium, Spain and the Netherlands. No other differences between the countries could be found.

### Determinants of positive mental health

**Figure 1:**
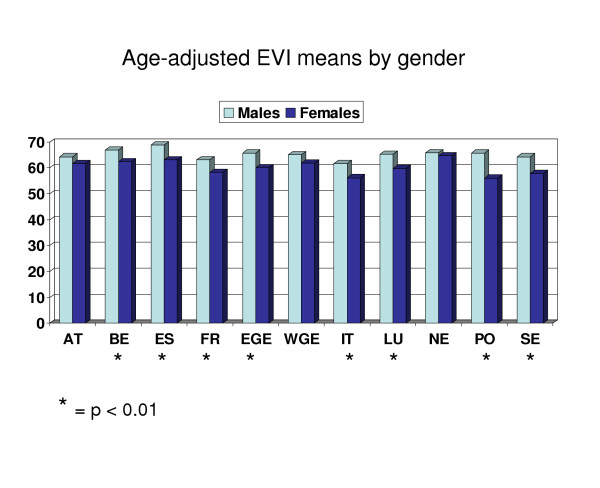
Age-adjusted EVI means by gender.

**Figure 2:**
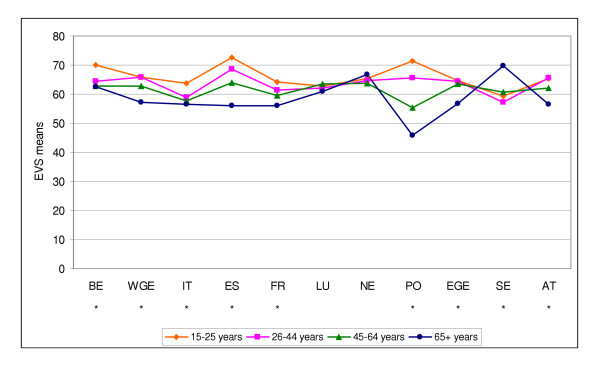
EVI means in different age groups (* = p < 0.01).

The EVI mean score was higher for men than for women in 8 of the 11 countries/regions; only in Austria, West Germany and the Netherlands this was not the case (Figure 1). Overall, the EVI mean score decreased with increasing age, but this was not true in all countries (Figure 2). In Sweden the reverse was true, and in Luxembourg and in the Netherlands there were no statistically significant differences between the four age groups.

Other variables which had a statistically significant association with the gender- and age-adjusted EVI mean score were family income (lowest in lowest income quartile), marital status (lowest in the widowed and separated), residency (lowest in large cities) and occupation (lowest in those on pension). The lowest income quartile had the poorest mental health status in all countries/regions, but for the other variables there were some variation across the countries/regions.

### Social support and mental health

**Figure 3:**
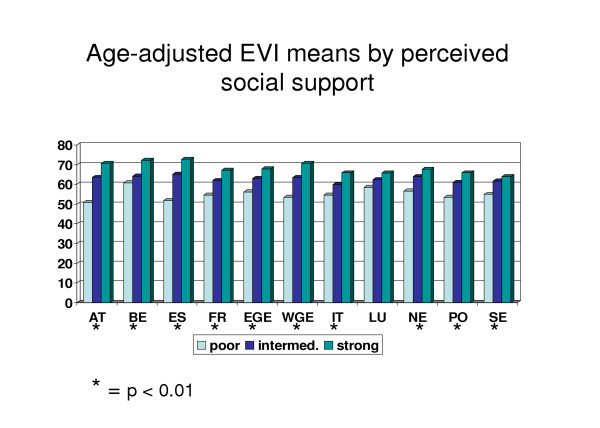
Age-adjusted EVI means by perceived social support.

**Figure 4:**
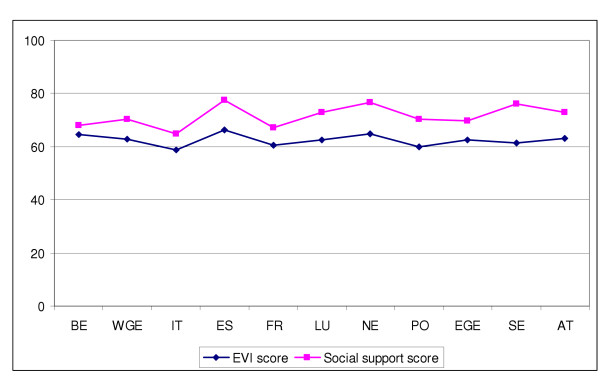
EVI mean and social support score by country.

Social support, which in itself could also be seen as an indicator for mental health, was strongly associated with EVI score (Figure 3). The same statistically significant pattern could be found in all countries except Luxembourg (mainly because of the small sample size from this country). A very interesting finding was that the social support mean score and EVI mean score followed systematically each other from country to country, as can be seen from Figure 4: High social support score in a country predicted high EVI score in that country and vice versa.

### Multivariate analysis of mental health

The mean EVI score was analysed by MANOVA to look at the importance of the different determinants in a multivariate context (Table 1). All variables, except age, had a statistically significant association with EVI mean score also in this analysis. As can be interpreted from the F-values, the most important determinants were social support, gender and income. However, one has to consider the fact that the variables included in the analysis explained only 11.6% of the total variance.

**Table 1:** MANOVA of the EVI mean

Variable	F value	p	Poorest mental health
Support	129.2	<.0001	Low social support
Gender	76.4	<.0001	Females
Income	33.5	<.0001	Lowest quartile
Marital status	7.2	<.0001	Widowed
Country	6.3	<.0001	Italy
Living area	5.1	<.0059	Large cities
Occupation	3.9	<.0037	Retired
Age	3.4	<.0164	65 years and over

## Discussion

### Methodological issues

There does not exist complete agreement among researchers about the concept and measurement of positive mental health [19]. Anyhow, the Energy and Vitality Index (EVI) has been regarded as a relevant and feasible measure for positive mental health, and it has, for example, been recommended by the development project 'Establishment of Mental Health Indicators for Europe' as an relevant indicator for mental health monitoring [20]. In this study, as in earlier studies, its reliability, as measured by the Cronbach's Alpha, was shown to be satisfactory.

In interpreting the results of this study, several reservations have to be made. One should be extremely careful especially when comparing the countries with each other. Despite the fact that the mental health indexes of the SF-36 are well-established and tested in several previous studies, and despite the instructed translation process in this study, the possibility of slight conceptual differences in the final translated questionnaires between countries cannot be fully excluded. The true validity of EVI in a multicultural context remains still uncertain in many regards.

The Eurobarometer interviews have been conducted by national opinion survey agencies, and at least a minimum level of expertise in interviewing can thus be expected. However, differences may well exist in the orientation of these agencies toward mental health questioning, leading to some additional variation between countries.

Although the Directorate-General Press and Communication of the European Commission has given instructions on how the sampling of the research subjects should be conducted, exact and detailed information on the sampling procedures in the different countries/regions is lacking. Therefore, we cannot be totally sure about the representativeness of the national samples. One additional problem is the low response rate in many of the participating countries/regions. Although countries with the poorest response rate were excluded from this study, there still were five countries where the response rate was lower than 60%.

Furthermore, the cultural differences most likely influence the act of interviewing to some extent. The interaction with the interviewer and the role of the participant, as well as the understanding and interpretation of terms and concepts by people from different cultural backgrounds may differ and cause variation between participating countries. Furthermore, cultural variation seems also to exist in experiencing and expressing the inner feelings and emotions.

### Positive mental health in Europe

The results of this study indicated differences in the state of positive mental health between the different EU Member States. The mean EVI score was relatively low in Italy, Portugal, France and Sweden and above the average in the Netherlands, Spain, Belgium and West Germany. As stated above, it is possible that these results are mainly due to methodological/cultural biases, but of course, they can also indicate true differences between the countries. Unfortunately, there are hardly any study available with which to compare the results from this study.

The World Database on Happiness [7] can provide some possibility for comparison, although the construct happiness and the positive mental health as defined in this study are by no means same thing. However, we can find some similar trends in the results. For example, low level of happiness has been measured in France, Italy and Portugal, whereas the Netherlands and Belgium show a score that is above the crude average for the respective ten countries. On the other hand, there also seem to exist evident inconsistencies: Spain has a low score in happiness in the database but high score in positive mental health in this study, and for Sweden the reverse is true. The conclusion, thus, could be that subjective experience of happiness can be seen as a relevant indicator for positive mental health in most countries, but for some countries (for example Spain and Sweden) this is not the case.

### Determinants of positive mental health

In this study the average EVI score was significantly higher in men than in women in 8 of the 11 countries/regions, indicating better mental health in men. Some studies have yielded similar results using sense of coherence as measure of psychological well-being [21,22,6] but in most of the studies no gender difference has been found. In the Australian Survey of Mental Health and Well-being women had higher life satisfaction than men [5]. On the other hand, better mental health in men is in concordance with the fact that most epidemiological surveys present for women higher prevalence of common mental disorders than for men. It is also likely that sense of coherence or life satisfaction are somewhat different measures than EVI, meaning that their direct comparison is not fully justified.

In 8 of the countries the mental health decreased with increasing age. In two countries (Luxembourg and the Netherlands) there were no significant differences between the age groups, but in Sweden the association was reverse: mental health increased with increasing age. Several studies have shown how sense of coherence increases with increasing age [22-24]. On the other hand, our study supports the finding from the Australian Survey, in which life satisfaction became less common with age in both sexes [5].

Other factors that were associated with poor mental health in this study were poor economic situation, being widowed or separated or living in a large city. All these factors have also be seen as determinants of mental disorders in most epidemiological studies. Several studies have also shown an inverse correlation between sense of coherence and socio-economic status.

The most interesting finding in this study was the strong association between mental health and social support. Strong link between social support and mental health has also been found in many other studies [25,26,4,6]. The most interesting finding of this study, however, was the evidence that the level of social support predicted the state of positive mental health in between-country comparisons. This supports the recommendation by the EU Mental Health Indicator project [15] to include measure of social support as an indicator for mental health.
